# Does community HIV combination prevention (HPTN 071 (PopART)) impact healthcare inequity?

**DOI:** 10.1093/eurpub/ckac131.008

**Published:** 2022-10-25

**Authors:** C Hansen, M Miraldo, K Hauck

**Affiliations:** 1 Business School, Imperial College London, London, UK; 2 School of Public Health, Imperial College London, London, UK

## Abstract

**Background:**

This research explores the impact of the HIV Prevention Trials Network (HPTN) 071 combination prevention (PopART) trial on horizontal inequity in Zambia and South Africa. Evidence suggests there is often inequality in healthcare utilisation in relation to socioeconomic characteristics, such as wealth. This paper is the first to address such distributional outcomes and inequities in a randomised trial setting.

**Methods:**

We utilise horizontal inequity as a key performance metric to make value judgements with regards to the distribution of healthcare utilisation in a health system. Additionally, we supplement this index by estimating the impact of PopART on inequality in healthcare utilisation and HIV prevalence, estimating concentration curves and indices.

**Results:**

We find a pro-rich inequity in healthcare utilisation, as ranked by wealth, before and after the trial is implemented. Pro-rich implies, for example, the 20% poorest make up less than 20% of healthcare utilisation. This suggests the trial enabled the wealthier subset of the population to take better advantage of accessing healthcare.

**Conclusions:**

Given the high prevalence of HIV in lower-income households in Zambia and South Africa, these results strengthen the case for interventions tailored to informing poorer households about the benefits of prevention and after-care.

**Key messages:**

• The PopART trial demonstrates as with any intervention, there is a risk of exacerbating an underlying inequity. Policies directed at these problems specifically may help alleviate such burdens.

• This paper shows one example of a distributional imbalance in the HIV population. Given the absence of work on RCT interventions, the findings may also be used to inform future trial design.

